# Brain Metastasis From Advanced-Stage Lung Carcinoma: Differentiating From Stroke and Exploring Treatment and Prevention Methods

**DOI:** 10.7759/cureus.79794

**Published:** 2025-02-27

**Authors:** Karilyn Sims, Natalie Saliba, Michele Goldhagen

**Affiliations:** 1 Medical School, Edward Via College of Osteopathic Medicine, Auburn, USA; 2 Hospital Medicine, Russell Medical Center, Alexander City, USA

**Keywords:** advanced-stage lung cancer, brain metastasis, non-small-cell lung carcinoma (nsclc), stroke, van scale

## Abstract

Metastasis is one of the most significant contributors to mortality and treatment-related morbidity in patients with advanced-stage lung cancer, as it is often considered incurable once discovered. Accordingly, a significant challenge with metastasis is identifying its progression early, as initial imaging may be negative while metastasis propagates undetected. Hence, there is a growing consensus that determining the optimal frequency and improving screening protocols for brain metastasis in patients with lung cancer are essential areas of research, as earlier detection could allow for prompt adjustments in treatment and/or prophylactic interventions, thereby potentially improving outcomes and reducing risks associated with more invasive procedures. Moreover, another difficulty in the diagnosis of brain metastasis can arise from its symptom overlap with strokes, often necessitating methods such as the use of the visual-aphasia-neglect assessment scale, as well as accounting for patient history and timeline of symptoms, with definitive diagnosis established through a head CT/MRI.

This report presents a patient with a history of a tobacco use disorder, upper esophageal stricture, and a one-year history of advanced stage III non-small-cell lung carcinoma who reported to the emergency department (ED) with concerns of neurological weakness where multiple lesions with vasogenic edema were discovered on head CT, suggestive of brain metastasis. The patient was admitted to the hospital, where he was treated with dexamethasone and subsequent MRI confirmed the diagnosis of brain metastasis. He was discharged home to begin whole brain radiation therapy with orders to temporarily hold his chemotherapy medication. The patient initially responded well, but most recent CT scans have indicated progression of metastasis to bone and gastrointestinal organs. Thus, earlier recognition of impending brain metastasis, especially in advanced-stage lung carcinoma, is a significant area of research, as timely detection can have decisive impacts on patient outcomes and possible interventions.

## Introduction

Lung cancer is the leading cause of cancer-related deaths in the United States, with metastasis being one of the most significant contributors to mortality in these patients [1]. Non-small-cell lung cancer (NSCLC) is the most prevalent type of lung cancer, and while it does progress slower than its small-cell counterpart, 40% of NSCLC cases already have some degree of metastasis by the time they are diagnosed [2,3]. The two most common sites of metastasis from the lungs are the brain and bones, and once metastasis occurs, a patient’s prognosis significantly decreases to a less than 10% five-year survival rate [4]. Typically, primary lung cancer will spread to regional lymph nodes in the chest first and then begin expanding to more distant lymph nodes and other sites of the body. The prognosis for NSCLC patients presenting with only regional lymph node metastasis is markedly better, with a five-year survival rate of 37%; meanwhile, NSCLC that has distant organ/site metastasis shows a five-year survival rate of 8% [5]. Hence, the spread to lymph nodes in the neck would indicate a transition from regional to distant metastasis and could be an early sign of the progression of cancer to the brain. Furthermore, studies have found that about 10%-25% of patients with lung cancer have brain metastases at diagnosis and another 40%-50% develop it during their disease process [6,7]. Therefore, improving current screening protocols for brain metastasis in patients with lung carcinoma is critical, especially in those with high-risk features and/or advanced-stage cancer.

In addition, one of the challenges in diagnosing brain metastasis is how closely related the symptoms are to strokes, which occur when blood carrying oxygen and nutrients to the brain is interrupted, causing transient or permanent neurological damage to one or more areas of the brain. Common symptoms of both include dizziness, numbness, headaches, unilateral/bilateral weakness, and problems with vision, speech, and/or spatial awareness.

Therefore, discerning between brain metastasis and a stroke can be difficult, especially with neurologic deficits. A few markers of differentiation include using the timeline of symptom presentation - strokes tend to occur suddenly, while metastasis is more progressive - as well as patient history and CT/MRI scans, which are defining for diagnosis [8]. While a complete neurological screening examination is superior, one screening tool that is commonly used in emergency settings is the visual-aphasia-neglect (VAN) scale, which is useful in testing for possible large-vessel occlusions that may affect a patient’s vision, impede one’s quality or ability to produce speech, and/or cause hemispatial neglect [9]. Therefore, if these areas are unaffected but the patient still presents with an extremity neurologic deficit, it is indicative to begin looking at other causes such as brain metastasis or strokes affecting smaller arteries. In any case, a definitive diagnostic test such as head CT would need to be performed.

## Case presentation

A 63-year-old African American male patient presented to the emergency department (ED) after his daughter brought him from home with concerns of weakness in his legs and left arm. As the patient was a poor historian due to minimal desire to answer questions and mild confusion, his daughter helped provide his history to the extent of her knowledge. He had presented to the ED the day before for a two-day history of generalized weakness and nausea, most likely due to the initiation of docetaxel two days prior for refractory complications with his one-year history of stage III NSCLC. When asked to compare the visits, the patient reported it was the same; however, nursing staff noted his weakness appeared to have increased, as he had ambulated into exam rooms upon his initial visit versus requiring wheelchair assistance on this day. During the initial visit, the patient was diagnosed with hypokalemia, for which he was given a potassium supplement along with ondansetron for nausea. A head CT was not performed, and the patient was discharged with instructions to come back if conditions worsened. After his discharge, the patient and his daughter spoke with his oncologist, who recommended he be seen at the ED again for a head CT.

The patient had at least a 30-pack-year history of smoking. His past medical history is significant for at least stage III NSCLC in the right upper lobe bronchus that had spread throughout the middle and lower lobes. At the time of lung cancer diagnosis 15 months ago, PET/CT scans also revealed a right supraclavicular node, right upper paratracheal node, multiple additional paratracheal nodes, and enlarged para-aortic nodes. The patient also had an upper esophageal stricture one year prior to ED presentation that was subsequently seen by a gastroenterologist and treated with dilatation during an esophagogastroduodenoscopy. The patient denied fever, chills, nausea, vomiting, diarrhea, constipation, or sick contacts, as well as headache, vision changes/loss, and difficulty swallowing. The patient also denied any dizziness or numbness but reported generalized weakness with increased unilateral weakness in the left upper extremity and mild weakness in bilateral lower extremities.

At the present ED visit, the patient’s vital signs included a temperature of 97.9°F, pulse of 103 beats/minute, blood pressure of 118/75 mmHg, respiration rate of 18 breaths/minute, and 98% oxygen saturation on room air. Upon physical examination, the patient was alert and oriented to person/place/time with a Glasgow Coma Score of 15. He was in no acute distress, had no obvious facial asymmetry, was normocephalic, and had no trauma; the trachea was midline with no tenderness; and extraocular movements were intact. His cardiovascular and gastrointestinal examinations were unremarkable; the patient’s lungs were clear to auscultation bilaterally with non-labored respirations. The patient had marked unilateral weakness in his left upper extremity and was unable to contest gravity, showing grip strength of 2/5 versus 5/5 strength in the right upper extremity. The patient also had mild weakness in bilateral lower extremities but was able to lift his legs off the bed against gravity.

**Figure 1:**
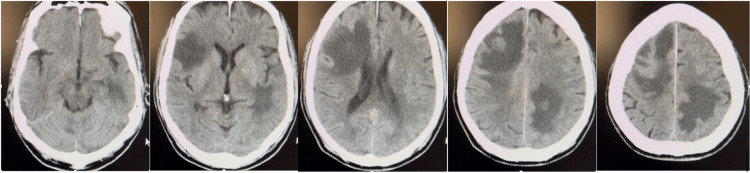
CT head images from left to right show multifocal intracranial lesions with vasogenic edema indicative of metastases, proceeding in a caudocephalad direction.

Initial labs revealed the patient had leukopenia, as shown in Table 1, with a white blood cell (WBC) of 1.12 K/μL, and his potassium level was still low but had increased since the previous ED visit. His COVID-19 and flu tests were all negative. Urine testing revealed trace blood and bacteria and was positive for cannabis. The patient’s non-contrast CT results revealed multifocal intracranial lesions with associated vasogenic edema with mild mass effect. These findings are most consistent with metastatic disease, as shown in Figure 1. The patient also had a CT angiography (CTA) performed, which revealed no flow-limiting stenosis or occlusion of major intracranial or cervical arteries. Multiple bilateral pulmonary nodules and a partially imaged right upper lobe mass were also seen, along with asymmetrically enlarged right level IIb nodes, suspicious for metastasis in this clinical setting. As such, the patient was diagnosed with CNS metastasis from stage IV NSCLC, hypokalemia, and bicytopenia.

**Table 1:** Patient lab values from the present emergency department (ED) visit revealed levels consistent with neutropenia and hypokalemia. Middle column (measured value): L: low; H: high

Laboratory test	Measured value	Reference range
White blood cell (WBC)	1.12 L	3.91-8.77 K/μL
Red blood cell (RBC)	3.80 L	4.18-5.48 M/μL
Hemoglobin (Hgb)	9.3 L	11.9-15.4 g/dL
Hematocrit (Hct)	30.2 L	38.2%-46.3%
Mean corpuscular volume (MCV)	79.5 L	80.0-93.8 fL
Mean corpuscular hemoglobin (MCH)	24.5 L	28.5-31.4 pg
Mean corpuscular hemoglobin concentration (MCHC)	30.8 L	31.9-34.8 g/dL
Platelet count	226	150-375 K/μL
Mean platelet volume (MPV)	9.5 L	9.7-13.9 fL
Neutrophil % (auto)	5.4 L	37.0%-80.0%
Lymphocyte % (auto)	47.3	15.0%-55.0%
Neutrophil # (auto)	0.06 L	1.5-7.0
Lymphocyte # (auto)	0.53 L	1.0-4.8 K/μL
Sodium	141.0	136-145 mmol/L
Potassium	3.3 L	3.5-5.1 mmol/L
Chloride	106.0	98-107 mmol/L
Carbon dioxide	29.00	23-29 mmol/L
Anion gap	6.0	5-15 mmol/L
Blood urea nitrogen (BUN)	12.0	8-20 mg/dL
Creatinine (Cr)	1.39 H	0.7-1.3 mg/dL
Glomerular filtration rate calculation (GFR)	57 L	>60
Glucose	104.0	70-105 mg/dL
Albumin	3.4 L	3.5-5.7 g/dL

**Figure 2:**
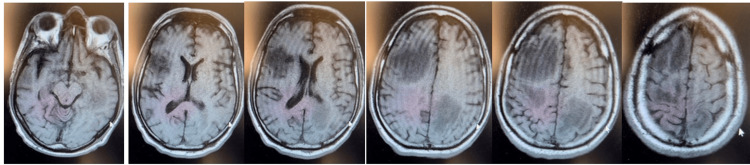
From left to right in a caudocephalad direction, brain MRI displays numerous intracranial lesions involving bilateral cerebral hemispheres. The largest lesions are located within the anterior right frontal lobe, left frontoparietal region, and left temporal lobe, with multiple smaller lesions scattered throughout. These lesions are accompanied by vasogenic edema with mild mass effect. The overall imaging findings are most consistent with CNS metastasis.

The patient was admitted to the hospital and started on dexamethasone 4 mg twice daily to minimize neurological symptoms and relieve intracranial pressure, along with continued treatment for hypokalemia. The patient was also put on neutropenic precautions with orders to start infectious protocol and broad-spectrum antibiotics if his temperature exceeded 100.4°F. Pantoprazole and enoxaparin sodium were initiated for gastrointestinal and deep vein thrombosis prophylaxis. During the patient’s hospital course, an MRI was performed, which confirmed multiple new brain metastases with vasogenic edema as shown in Figure 2. The interpreting radiologist also cited associated susceptibility artifacts in the larger lesions on diffusion imaging, suggesting the presence of possible hemorrhagic components. The patient’s oncologist was consulted and confirmed metastasis to the brain as well as bone, with plans to increase dexamethasone to 6 mg every eight hours and discontinue enoxaparin sodium due to the possible hemorrhages and instead start mechanical prophylaxis with thromboembolic deterrent stockings and sequential compression devices for venous thrombosis risk. As the patient was not exhibiting any seizure activity, the oncologist determined that seizure prophylaxis was not required at the time. The patient chose to be discharged home and subsequently began whole brain external radiation therapy dosed at 30 Gy over 10 fractions while holding docetaxel. Per outpatient records, the patient had been tolerating radiation therapy well and docetaxel was re-initiated, but the patient’s most recent CT scans revealed new developments in the liver and possibly pancreas, indicating refractory disease to second-line docetaxel. The patient’s oncologist has since discussed a third-line option, as well as recommended hospice care for the patient and his family to decide on together.

No osteopathic manipulative medicine (OMM) treatments were indicated or performed in the ED, as the patient was not having breathing difficulties, headache, nor visible edema. Additionally, no OMM treatments were recommended as both lymphatic techniques and high-velocity low-amplitude (HVLA) are contraindicated in bony metastasis, and suboccipital release is contraindicated due to the concern for hemorrhage on MRI.

## Discussion

Metastasis occurs when primary tumor cells migrate via lymphatic or hematologic routes to locate their preferred destinations and form communication and extravasation. These migrant tumor cells will then use the resultant inflammation from their invasion to establish their own metastatic niche within foreign tissue [10]. These lesions put pressure on the surrounding brain tissue, which changes its function and causes neurologic symptoms/deficits that are relative to the location of the lesion(s) [11]. As previously mentioned, these symptoms can make it difficult to differentiate whether the cause is from metastatic lesions or a cerebrovascular accident. Hence, the VAN assessment scale can be utilized to quickly arrange differential diagnoses in emergency settings, as well as analyze relative factors in the patient’s timeline of symptoms and medical history. Nonetheless, the definitive diagnosis must be made with CT or MRI. As seen in Figure 1, the patient’s lesions avoided sections of the brain most associated with vision, aphasia, and neglect, which explains why our VAN assessment did not lend support to major vascular stroke as the patient presented with no speech slurring or facial droop, no visual field changes/complaints of vision loss or blurring, nor spatial neglect. Moreover, there are several key features used to discern between a stroke and brain tumor/metastasis on CT imaging. For instance, hemorrhagic strokes typically present as hyperdense areas without surrounding vasogenic edema, while a brain tumor is characterized by the presence of edema (as illustrated in Figure 1) and is more likely to localize within the gray and white matter junction of the cerebral cortex, exhibit well-demarcated margins, and lack direct association with specific brain arteries. The presence of multiple lesions raises suspicion of metastasis [8].

Currently, brain metastasis is widely considered incurable, with a mostly palliative treatment approach consisting of surgery and/or radiation therapy. When brain metastasis is diagnosed, treatment often involves surgical debulking and/or excision (usually just in cases of one or two lesions) and/or whole brain radiation therapy (WBRT) along with a temporary hold on the patient’s chemotherapy - as seen with docetaxel in our patient - due to its inability to penetrate the blood-brain barrier, rendering it ineffective in treating brain metastasis. Thus, there are studies currently being conducted investigating the coupling of chemotherapy agents with pharmaceuticals that are able to cross the blood-brain barrier, such as lapatinib, which has been shown to have better uptake compared to others [7].

Therefore, given the limited effectiveness of chemotherapy, which remains a subject of ongoing research, many studies are increasingly focused on the prevention of metastatic formation rather than post-diagnostic interventions. While still under investigation, literature shows that prophylactic cranial intervention (PCI) using WBRT has a reductive impact on the incidence of brain metastases in NSCLC, although there is no significant overall survival benefit [7]. This study also explores the role that radioprotection/sensitization could have in preventing cognitive decline from WBRT, while also further investigating the preventive role of pharmaceutical therapies through the employment of molecular pathways and brain-permeable inhibitors [7]. As the neuroinflammatory environment is one of the key factors used in the formation of metastatic niches, the inhibition of this response could be significant in both the protection from WBRT-induced cognitive decline and the prevention of metastasis.

While currently it is recommended to conduct initial brain MRI/CT scans with known metastatic stage III NSCLC, there is no definite consensus on how frequently these screenings should be performed. Consequently, research has shifted more toward earlier detection of brain metastasis, as it can propagate unnoticed for years if the patient remains asymptomatic. This was seen in our patient, whose initial MRI was performed two months after his NSCLC diagnosis proved unremarkable. However, without symptoms prompting repeat brain scans, metastatic spread was discovered throughout the brain when a follow-up CT (see Figure 1) was performed roughly one year later upon presentation to the ED with his concern of neurological weakness. According to literature, around 20%-40% of patients with NSCLC will develop brain metastases during their disease course, many asymptomatically and with initial negative MRIs, which potentiates the question of how often these patients should be screened with imaging [7,12,13].

Therefore, studies are currently being performed to identify patients at high risk of developing brain metastasis, as there is a consensus that the earlier providers can recognize signs of impending metastasis, the earlier the appropriate treatment(s) can be initiated, which could also spare the patient from more invasive (surgical) and/or toxic (systemic) therapies [12,14]. One study developed a predictive model using comprehensive clinical and genomic factors to aid in determining the risk of brain metastasis in lung cancer patients and found that the histology, stage, and central location of primary tumors were significant drivers in dissemination [14]. A notable feature in this patient’s case is his history of an upper esophageal stricture, which occurred around the same time as his diagnosis. As per previous chart records, it was found that initial CT/PET scans performed after his initial diagnosis revealed the patient had numerous enlarged, possibly necrotic lymph nodes in the superior mediastinum and paratracheal chain (among others). Therefore, it was presumed that these enlarged paratracheal lymph nodes could be causing the patient’s esophageal obstruction by impinging on the nearby organ and, as mentioned in the introduction, be an early sign of distant metastasis because it suggests that the cancer was no longer limited to regional spread within the chest but had begun its ascension to the brain through the expansion to cervical lymph nodes.

Furthermore, the predictive model study also noted that the patients who were identified as “high-risk” and underwent more frequent brain MRIs showed smaller metastases and reduced rates of surgical intervention compared with those who did not have as many MRIs performed [14]. This supports the notion that patients, especially those at high risk of metastasis, will likely benefit from more aggressive brain MRI or head CT surveillance earlier in the disease process, which could lead to better outcomes and less invasive treatment options [12]. For instance, clinicians might consider regular brain imaging at specific intervals depending on initial diagnostic staging - such as every three to six months for advanced stages or those classified with high-risk features and every six to 12 months for patients with earlier-stage diagnoses.

## Conclusions

In conclusion, as brain metastasis can frequently be undetected on initial imaging and develop asymptomatically over time, this case stresses the importance of establishing definitive screening protocols for patients with advanced-stage lung carcinoma at high risk for metastasis, as waiting for symptom onset is often associated with increased morbidity and more invasive treatment options. Since brain metastasis continues to be considered incurable and chemotherapy options disappointing, research is increasingly shifting toward earlier detection through the identification of high-risk patients using predictive models, recognition of early signs of metastasis such as lymph node dissemination, and the employment of prophylactic cranial irradiation in the form of WBRT. This case underscores the potential impact of routine brain imaging in enabling earlier detection of impending metastasis, which would allow healthcare providers to intervene more promptly, facilitating adjustments to treatment plans that can enhance patient prognosis and reduce the morbidity and invasiveness of necessary interventions.
